# Time to Flexible Sigmoidoscopy or Colonoscopy in Patients Admitted With Ulcerative Colitis Has Decreased From 2012 to 2018

**DOI:** 10.1093/crocol/otab080

**Published:** 2021-12-05

**Authors:** Aman S Bali, Michael F Picco, Jana G Hashash, Francis A Farraye, Paul T Kröner

**Affiliations:** 1 Department of Internal Medicine, Mayo Clinic, Jacksonville, Florida, USA; 2 Division of Gastroenterology and Hepatology, Mayo Clinic, Jacksonville, Florida, USA

**Keywords:** ulcerative colitis, flexible sigmoidoscopy, colonoscopy, timing

## Abstract

**Background:**

Early endoscopic evaluation of patients with ulcerative colitis (UC) enables assessment of disease activity and accurate diagnosis based on exclusion of other similarly presenting conditions including infections. Early endoscopy is also associated with improved outcomes of patients with active UC. The aim of this study was determining temporal trends in endoscopy in patients with UC over a 7-year period from 2012 to 2018.

**Methods:**

Retrospective cohort study using the National Inpatient Sample 2012–2018. Patients admitted with ICD-9–10 principal codes for UC were included. Early endoscopy using flexible sigmoidoscopy (FS) or colonoscopy was defined as performed within 48 hours of admission. The primary outcome was trends in endoscopy timing. Secondary outcomes were inpatient morbidity, mortality, length of stay (LOS), and hospitalization charges/costs comparing patients undergoing early vs nonearly endoscopy using multivariable regression.

**Results:**

Of 222 460 patients hospitalized with UC, 5900 (2.7%) underwent FS and 43 345 (19.5%) underwent colonoscopy. The rate of endoscopy increased from 3.9% (2.3% early) to 39.3% (23.3% early) from 2012 to 2018 (*P* < .01). Early endoscopy was associated with statistically significant decreased mortality, shock, multiorgan failure, and intensive care unit odds, as well as decreased resource utilization and LOS.

**Conclusions:**

In patients hospitalized with UC, early endoscopy rates were low but performed more frequently from 2012 to 2018. This may reflect increasing awareness of improved outcomes from earlier disease staging and/or diagnosis. Early endoscopy was associated with decreased resource utilization and hospitalization-related outcomes, highlighting the importance of early endoscopy in patients admitted with UC.

## Introduction

Inflammatory bowel disease (IBD) represents a significant healthcare expenditure burden in an era of value-based care, affecting more than 6.8 million people worldwide with a rising incidence in newly industrialized countries.^1–5^ Ulcerative colitis (UC) constitutes a majority of IBD cases and contributing to these challenges, the optimal management of patients hospitalized with UC is complex owing to unique management considerations in a patient population that is often immunocompromised and at risk of overlapping infections, including cytomegalovirus (CMV) colitis.^6^ Given these complexities, guidelines from the Canadian Association of Gastroenterology (CAG) as well as the British Society of Gastroenterology (BSG) recommended early endoscopic evaluation of patients hospitalized with UC to help determine symptom etiology and disease severity.^7,8^ Using the National Inpatient Sample (NIS), Obi et al provided additional evidence that early endoscopy, defined as flexible sigmoidoscopy (FS) or colonoscopy within 2 days of hospitalization, was associated with favorable patient and hospitalization outcomes.^6^ This included decreased rates of colectomy, mortality, length of stay (LOS), and costs associated with index hospitalization.^6^ Since that time, additional guidelines from the American College of Gastroenterology (ACG) and the BSG have supported early endoscopic evaluation of patients hospitalized for UC.^3,9^ However, it remains to be seen if these guidelines have affected the temporal trends of when endoscopy is performed in current practice. The goal of this study, therefore, was to determine the temporal trends in the timing of FS/colonoscopy in relation to day of admission for patients hospitalized with UC in the past 7 years using the latest nationally representative data from the NIS (2012–2018). Secondary outcomes included assessment of patient-specific and hospitalization-associated outcomes, including rates of mortality, shock, multiorgan failure, intensive care unit (ICU) admission, LOS, and costs/charges of hospitalization.

## Materials and Methods

### Study Design and Data Source

This retrospective cohort study used all information extracted from the Nationwide Inpatient Sample (NIS) years 2012–2018, which is the largest publically available, inpatient dataset in the United States. This dataset is a 20% stratified sample of all hospital admissions from more than 4000 nonfederal acute care hospitals across more than 40 states of the United States. The dataset includes a principal diagnosis (the primary diagnosis at discharge), and well as up to 29 secondary diagnoses. Up to 15 procedural codes are also included in the dataset, which represent all procedures performed during the hospital admission. The dataset contains information about the patient’s length of hospitalization, and total hospitalization charges.

### Study Population

All patients with principal diagnostic codes for UC (556.x and K51.xxx) were identified from within the database and included in the study. UC disease classification, as coded by the International Classification of Diseases, Clinical Modification (ICD-CM) changed from its 9th to 10th revisions during the study period, care was exercised in identifying and matching each diagnostic and procedural code to its corresponding code for the timeframe. Patients with ICD-9/10 CM procedural codes for FS (45.24 and ODJD8ZZ) and colonoscopy (45.23, 0DJD8ZZ, 0DBE8ZX, 0DBF8ZX, 0DBG8ZX, 0DBH8ZX, 0DBK8ZX, 0DBL8ZX, 0DBM8ZX, and 0DBN8ZX) were identified within the patient cohort with associated UC. Furthermore, the timing of performing FS or colonoscopies was substratified into “Early” and “Not Early,” depending on whether the procedure was performed within 48 hours of admission. The “Not Early” cohort included both patients undergoing endoscopy on hospital day 3 or later and patients no undergoing any endoscopic procedure. Elective admissions were excluded from analysis using the database modifier identifying admissions as elective.

### Variable Definition

Included patient characteristics were demographics (ie, age, gender, ethnicity, median income based on zip code, and insurance type). Hospital characteristics included hospital region, teaching status, hospital bed size (number of short-term acute care beds set up and staffed in a hospital), and hospital urban/rural location. Each of the included patient’s vital status at the end of the hospitalization (living vs dead) was documented, as were the total days of hospitalization (LOS) and total hospitalization costs (dollar amount collected from patient/insurance) and charges (dollar amount set as price for services). To control for patient comorbidities, the Deyo adaptation of the Charlson Comorbidity Index was used, which is a validated tool for large database analysis.^10^

### Aims

The primary aim was to determine the trends in the timing of FS and colonoscopies in patients with UC along the study period. Secondary outcomes were determining the odds of early FS or colonoscopy, inpatient mortality/morbidity odds, hospital LOS, and total hospitalization charges and costs. Morbidity was measured by the occurrence of shock, multiorgan failure, and ICU admission.

### Statistical Analysis

Discharge-level weights published by the HCUP were used to estimate the total number of patients with UC who underwent FS and colonoscopy in the study period. Fisher’s exact test was used to compare proportions and analysis of variance was used to compare means. To examine associations between FS/colonoscopy and the outcomes of interest in patients with UC, adjusted odds ratios (aORs) and adjusted means comparing the year 2018 to the year 2012 were obtained with a multivariate logistic regression model. The model was built by examining variables that have been associated with the outcomes on prior studies, followed by the conduction of a univariate regression analysis to determine the magnitude of the unadjusted association. If there was significant association on univariate analysis, as demonstrated by an association with a generous *P* value of <.10, they were included in the multivariate model. Finally, the multivariate model adjusted for age, gender, ethnicity, insurance type, median income in patient zip code, Charlson Comorbidity Index, hospital region, urban location, teaching status, and hospital bed size. All statistical calculations were conducted using STATA, Version 14 (StataCorp LP).

### Ethical Considerations

This study uses retrospective, cross-sectional, deidentified data from the NIS that is publically available. The our local Institutional Review Board determined this study was exempt from the requirement for review.

## Results

Between 2012 and 2018, a total of 222 460 patients with a principal diagnosis of UC who underwent nonelective admission for UC were identified within the dataset and included in the study. Among these patients 5900 (2.7%) underwent FS and 43 325 (19.5%) underwent colonoscopy. The mean age of patients undergoing early endoscopy was of 44.9 years (*P* < .01) and 52.7% of these patients were women (*P* = .91). In 2012, 1155 patients (3.9%) underwent endoscopy overall with 2.3% of patients undergoing early endoscopy compared to 13 115 patients (39.3%) undergoing endoscopy overall in 2018 with 23.3% of patient undergoing early endoscopy (*P* < .01). Further information regarding the study cohort is included in Table 1.

**Table 1.** Study population summary comparing cohort undergoing no FS/colonoscopy to cohort undergoing procedure, with *P* values displayed.

	No early endoscopy	Early endoscopy	*P*
Age (years)	47.3	44.9	<.01
Female (%)	52.8	52.7	.91
Ethnicity (%)			.02
Caucasian	69.1	69.6	
African American	13.4	12.0	
Hispanic	11.1	13.0	
Asian	2.2	2.0	
Other	2.2	3.7	
Median income (%)			.07
$1–37 999	25.6	23.4	
$38K–47 999	24.1	24.8	
$48K–63 999	26.0	25.7	
>$64 000	24.4	26.1	
Charlson Index (%)			<.01
0	64.7	64.6	
1–2	25.1	27.3	
>3	10.2	8.0	
Insurance (%)			<.01
Medicare	29.5	24.2	
Medicaid	18.5	18.6	
Private	43.1	46.8	
Self-pay	5.5	6.2	
Other	3.5	4.2	
Region (%)			<.01
Northeast	21.6	21.0	
Midwest	21.8	22.4	
South	37.8	34.7	
West	18.9	21.9	
Urban location (%)	95.2	95.7	.28
Teaching hospital	72.9	73.0	.86
Hospital bed size (%)			.16
Small	16.5	18.1	
Medium	28.6	27.6	
Large	55.0	54.3	

Abbreviation: FS, flexible sigmoidoscopy.

In crude univariate analysis comparing UC patients undergoing early endoscopy (FS or colonoscopy within 2 days) to those not undergoing early endoscopy (including either no endoscopy or endoscopy hospital day 3 or later), patients undergoing early endoscopy had lower total hospitalization costs ($11 740 vs $19 045, *P* < .01) and total hospitalization charges ($46 404 vs $75 581, *P* < .01). Additionally, patients undergoing early endoscopy had lower overall LOS (4.8 vs 9.1 days, *P* < .01) compared to patients not undergoing early endoscopy. The total rate of performance of FS/colonoscopy increased from 1155 out of 29 615 admissions (3.9%) in 2012 to 13 115 out of 33 345 admissions (39.3%) in 2018 (*P* < .01). Comparing patients undergoing endoscopy in teaching institutions vs nonteaching institutions, 73.3% underwent colonoscopy at teaching centers vs 26.7% of patients at nonteaching centers, while 69.9% of patients underwent FS at teaching vs 30.1% of patients at nonteaching centers (*P* < .05). These results of these analyses are summarized in Table 2 and Supplementary Table 1.

**Table 2.** Crude means comparing total hospital costs, hospitalization charges, and length of hospitalization in patients with UC undergoing early flexible sigmoidoscopy/colonoscopy compared to patients who underwent nonearly flexible sigmoidoscopy/colonoscopy.

Variable	No early endoscopy	Early endoscopy	*P*
Costs	$19 045	$11 740	<.01
Charges	$75 581	$46 404	<.01
LOS (days)	9.06	4.84	<.01

Abbreviations: LOS, length of stay; UC, ulcerative colitis.

In adjusted multivariable analysis, early FS/colonoscopy was associated with a statistically significant decrease in odds of shock (aOR 0.43 [0.15–0.61], *P* < .01), multiorgan failure (aOR 0.70 [0.60–0.81], *P* < .01]), ICU admission (aOR 0.46 [0.31–0.70], *P* < .01), and mortality (aOR 0.30 [0.15–0.61], *P* < .01). Additionally, early FS or colonoscopy was associated with a statistically significant decrease in resource utilization, including decreased additional costs (mean [95% CI] −$7578 [−$8439 to (−$6718)], *P* < .01) and additional charges per hospitalization (mean [95% CI] −$30 304 [−$33 733 to (−$26 875)], *P* < .01) as well as decreased additional LOS (mean [95% CI] −4.3 days [−4.6 to (−4.0)], *P* < .01). Overall results from multivariable analysis are shown in Table 3.

**Table 3.** Adjusted odds ratio of early FS/colonoscopy and adjusted means for selected variables comparing early flexible sigmoidoscopy/colonoscopy to nonearly FS/colonoscopy in patients with ulcerative colitis.

Variable	Adjusted OR	95% CI	*P*
Mortality	0.30	0.15 to 0.61	<.01
Shock	0.43	0.29 to 0.64	<.01
ICU admission	0.46	0.31 to 0.70	<.01
Multiorgan failure	0.70	0.60 to 0.81	<.01
Variable	Adjusted mean	95% CI	*P*
Additional costs	−$7578	−$8439 to −$6718	<.01
Additional charges	−$30 304	−$33 733 to −$26 875	<.01
Additional LOS (days)	−4.28	−4.59 to −3.97	<.01

Abbreviations: FS, flexible sigmoidoscopy; ICU, intensive care unit; LOS, length of stay; OR, odds ratio.

## Discussion

In this cross-sectional analysis, nationally representative data from the 2012–2018 NIS was used to identify patients hospitalized with principal diagnosis related to UC and assess trends in the timing of endoscopy (FS or colonoscopy). Secondary outcomes related to hospital LOS, resource utilization, and medical complications were assessed and compared across groups receiving early endoscopy (within 48 hours of admission) vs delayed endoscopy. Over the 7-year study period, the overall rate of performance of endoscopy remained low in UC-related hospitalizations. However, from 2012 to 2018 the rate of the early endoscopy increased significantly for both FS and colonoscopy. We demonstrated that early endoscopy was associated with a statistically significant decrease in hospital LOS, hospitalization costs/charges, and medical complications including both shock, multiorgan failure, ICU admission, and mortality.

Our findings that FS or colonoscopy occurring within 48 hours of hospitalization happened more often from 2012 to 2018 may represent increased recognition of improved outcomes and adoption of national guidelines. Recent guidelines from governing organizations including the ACG, CAG, and BSG have all recommended the use of “early” endoscopy in patients hospitalized with UC.^3,8,9^ In a 2012 publication of consensus statements, the CAG recommended FS with biopsies “as soon as possible” after a patient is admitted to the hospital.^8^ In 2019 guidelines, the BSG recommended that all patients presenting with possible acute severe ulcerative colitis (ASUC), defined as the presence of at least 6 bowel movements daily and any systemic sign of toxicity (fever, tachycardia, anemia, or elevated inflammatory markers), have urgent inpatient assessment including FS, laboratory studies, and imaging.^9^ This was an update to their previous 2011 guidelines at called for FS with biopsy for CMV within 72 hours (and ideally 24 hours).^7^ ACG guidelines in 2019 also specified a timeframe for endoscopy, stating that patients with ASUC should undergo FS within 72 hours of admission and preferably within 24 hours.^3^

However, other guidelines from the European Crohn’s and Colitis Organization (ECCO) recommend FS in patients admitted with ASUC but provide no explicit recommendations regarding the timing of endoscopy.^11^ The American Gastroenterological Association (AGA) recommendations regarding the management of hospitalized patients with ASUC provide guidance limited only to the choice of medical therapy with no explicit recommendations regarding endoscopy.^12^ These discrepancies in the recommendations of different governing bodies may reflect the uncertainty of clinicians overall, as our analysis showed that even in 2018, the total rate of performance of early FS or colonoscopy was only 39.3% of UC-related hospitalizations. Of note, these recommendations pertain to patients with ASUC who may overlap with but do not fully reflect our study population due to lack of inclusion of patient-level vital sign and laboratory study data in NIS.

Our study also demonstrated that early endoscopy was also associated with better hospitalization-related outcomes, including shorter LOS, lower costs/charges, and lower rates of medical complications. These findings are consistent with a prior analysis by Obi et al using data from the NIS, which also demonstrated that early endoscopy in patients with moderate UC severity was associated with lower in-hospital mortality and rates of colectomy.^6^ These findings may be attributable to the added diagnostic utility of early endoscopy as well as possible selection bias, as clinicians may perform endoscopy earlier during hospitalization in lower acuity patients who are more likely to have shorter hospitalizations, lower costs, and lower mortality.

Supporting recommendations for early endoscopy, multiple studies have demonstrated the safety of both FS and colonoscopy in patients presenting with ASUC.^13,14^ Additional utility of early FS/colonoscopy includes endoscopic evaluation of the severity of inflammation and confirmation of diagnosis, both through gross appearance of the colonic mucosa and biopsy for histopathology. Studies have demonstrated that ASUC can have a high degree of overlap with CMV infection, especially in steroid-refractory UC.^15,16^ Early confirmation of a diagnosis of CMV may permit more timely initiation of appropriate medical therapy, including antiviral agents. Importantly, endoscopic evaluation may even provide additional information related to prognosis. In retrospective analyses, severe endoscopic lesions were associated with nonresponse to corticosteroids and increased requirement for colectomy.^17–19^ Early endoscopy may thus permit more aggressive management including initiation of biologic agents and surgical consultation.

Although our study demonstrates a significant temporal trend in the rates and timing of endoscopy in patients hospitalized with UC, it should be interpreted with consideration of its limitations. First, the nature of the NIS database allows only for the execution of a retrospective analysis, which has its inherent limitations. Second, the database’s administrative nature makes it prone to miscoding bias or missing codes.^20^ Specifically, administrative datasets and retrospective studies only allow identification of correlation between variables, which does not (and should not) imply causation. However, ICD-CM codes have been shown to have a high specificity and sensitivity when used to study gastrointestinal diseases.^21^ Next, the precise severity of the patient’s disease activity cannot be elicited accurately from the dataset. Nevertheless, elective admissions were excluded and only the patients with principal diagnosis of UC were included in order to identify disease that was severe enough to require hospital admission. Despite being able to identify which patients underwent endoscopic procedures, the endoscopic findings and potential histologic findings are not able to be determined from this dataset. Also, the NIS does not contain information regarding use of medications and laboratory data. Lastly, readmissions are unable to be tracked within the database, as there are no patient-specific codes. Finally, a limitation of our analysis may be selection bias since sicker patients could be less likely to undergo endoscopy, leading to the appearance that endoscopy is associated with better outcomes. However, our analyses focusing on early endoscopy vs nonearly endoscopy may not be subject to this same degree of selection bias since only patients stable enough to undergo procedures are being compared.

Based on these limitations, further investigations into the timing of endoscopy in ASUC may gain benefit from being designed in a prospective, randomized manner. This will enable better attribution of causation rather than simply correlation. Additionally, inclusion of patients outside of the United States as well as laboratory and endoscopy findings may enable greater assessment of prognosis when analyzing which patients will benefit most from early endoscopy. Finally, further investigation is needed to understand why the overall rate of early FS/colonoscopy remained low at 39.3% even in 2018.

Overall, our analysis demonstrates national trends in endoscopy that may reflect increasing awareness of the favorable outcomes associated with early endoscopy in patients hospitalized with ASUC as well as more widespread adoption of national guidelines. Our study also redemonstrates the benefits of early endoscopy through favorable hospitalization-related outcomes including shorter LOS, lower hospitalization cost/charges, and lower rates of medical complications including shock, multiorgan failure, ICU admission, and mortality.

## Conclusion

In summary, analysis of NIS showed that in patients hospitalized with UC, early FS or colonoscopy performed within 48 hours of admission happened more often from 2012 to 2018. While the overall rate of early endoscopy remained low even in 2018, these temporal trends may reflect increased recognition of the benefits of early endoscopy and adoption of national guidelines.

## Supplementary Material

otab080_suppl_Supplementary_Table_S1

## Data Availability

Data underlying this manuscript were collected from the Healthcare Utilization Project (HCUP) National Inpatient Sample (NIS) encompassing the years 2012–2018. These data are publically available for purchase following completion of a Data Use Agreement and can be accessed at https://www.hcup-us.ahrq.gov/nisoverview.jsp.
