# Supplementary material for: Time to Flexible Sigmoidoscopy or Colonoscopy in Patients Admitted With Ulcerative Colitis Has Decreased From 2012 to 2018
Source: Crohns Colitis 360. 2021 Dec 5;3(4):otab080. doi: 10.1093/crocol/otab080 (PMC9802204; doi:10.1093/crocol/otab080)
Supplement: otab080_suppl_Supplementary_Table_S1 [file otab080_suppl_supplementary_table_s1.docx]

|  | **No Endoscopy** | **Endoscopy** |
| --- | --- | --- |
| Flexible sigmoidoscopy       Teaching       Non-teaching | 65.59%  34.42% | 69.92%  30.08% |
| Colonoscopy       Teaching       Non-teaching | 63.86%  36.14% | 73.27%  26.73% |

Supplemental Table 1 - Crude univariate analysis comparing the percentage of patients undergoing both flexible sigmoidoscopy and colonoscopy at teaching vs non-teaching hospitals.
